# Healthcare Costs by Comorbidity Patterns in Lung Cancer Patients

**DOI:** 10.3390/cancers17162682

**Published:** 2025-08-18

**Authors:** Alessandra Buja, Massimo Rugge, Marcello Di Pumpo, Manuel Zorzi, Federico Rea, Ilaria Pantaleo, Giovanna Scroccaro, Pierfranco Conte, Leonardo Rigon, Giorgio Arcara, Giulia Pasello, Valentina Guarneri

**Affiliations:** 1 Department of Cardiologic, Vascular and Thoracic Sciences, and Public Health, University of Padova, 35131 Padova, Italy; federico.rea@unipd.it (F.R.); ilaria.pantaleo@unipd.it (I.P.); 2 Department of Medicine (DIMED)—Pathology Unit, University of Padova, 35128 Padova, Italy; massimo.rugge@unipd.it; 3 Department of Life Sciences and Public Health, Università Cattolica del Sacro Cuore, 00168 Rome, Italy; 4 Department of Prevention, AULSS6 Euganea, 35131 Padua, Italy; 5 Veneto Tumor Registry (RTV), Azienda Zero, 35100 Padova, Italy; manuel.zorzi@azero.veneto.it; 6 Coordinamento Regionale per le Attività Oncologiche (CRAO), Regione Veneto, 30100 Venezia, Italy; giovanna.scroccaro@regione.veneto.it; 7 IRCCS San Camillo Hospital, 30126 Venice, Italy; pierfranco.conte@hsancamillo.it (P.C.); leonardo.rigon@hsancamillo.it (L.R.); giorgio.arcara@hsancamillo.it (G.A.); 8 Department of Neuroscience, University of Padua, 35122 Padua, Italy; 9 Department of General Psychology, University of Padua, 35122 Padua, Italy; 10 Oncologia Medica 2, Istituto Oncologico Veneto, Istituto di Ricovero e Cura a Carattere Scientifico, 35127 Padova, Italy; giulia.pasello@unipd.it (G.P.); valentina.guarneri@unipd.it (V.G.); 11 Department of Surgery, Oncology and Gastroenterology, University of Padova, 35138 Padova, Italy

**Keywords:** healthcare services, health economics, lung neoplasm, clusters

## Abstract

**Introduction:** Lung cancer imposes a substantial economic burden on patients, healthcare systems, and societies due to its high prevalence and costs associated with diagnosis, treatment, and palliative care. Comorbidities in lung cancer patients can further complicate clinical management and increase healthcare utilization. This study investigated the impact of comorbidity patterns on healthcare costs in patients with lung cancer. **Methods:** A cohort of 1540 lung cancer patients in the Veneto region of Italy was divided into five groups based on comorbidity burden using latent class analysis: no comorbidities, only one comorbidity, and specific comorbidity classes (Class 1: cardiovascular, respiratory, and endocrine diseases; Class 2: multiorgan diseases; Class 3: socio-multifactorial neuro conditions). Using administrative data, both overall healthcare costs and lung cancer-specific costs were analyzed over three years. **Results:** Patients with one comorbidity class had the highest overall costs over three years from diagnosis (USD 52,039) and the highest lung-specific costs (USD 47,804). In contrast, patients in the Cardiovascular-Respiratory and Endocrine class incurred the lowest overall costs (USD 38,447). Additionally, they had the lowest lung case-specific costs (USD 33,425) over the same three-year period from diagnosis. Higher costs for inpatient medications were observed in patients without any comorbidities or with at most one. **Conclusions:** The findings emphasize the significant effect of comorbidity patterns on resource use in lung cancer patients. Considering comorbidity profiles is essential for economic assessments and healthcare planning, as it allows for better resource allocation and supports personalized treatment strategies.

## Simple Summary

This study examined the impact of comorbidity patterns on healthcare costs for lung cancer patients over a 3-year period after diagnosis, using data from 1540 patients in the Veneto region of Italy. Patients were categorized into five groups based on comorbidity burden using latent class analysis: no comorbidities, one comorbidity, and three distinct comorbidity classes (cardiovascular/respiratory/endocrine; multiorgan diseases; socio-multifactorial neuro conditions). Patients with one comorbidity class had the highest overall 3-year costs (EUR 52,039) and lung cancer-specific costs (EUR 47,804). The cardio-vascular/respiratory/endocrine comorbidity class had the lowest overall costs (EUR 38,447) and lung cancer-specific costs (EUR 33,425). Higher inpatient medication costs were seen in those without comorbidities or with just one comorbidity. In the adjusted analysis, the socio-multifactorial neuro conditions class was associated with significantly higher overall costs compared to no comorbidities. The findings highlight the substantial impact different comorbidity profiles can have on healthcare costs and resource utilization for lung cancer patients. Considering comorbidities is important for economic as-assessments, healthcare planning, and developing personalized treatment strategies.

## 1. Introduction

Lung Cancer (LC) is a leading cause of cancer-related morbidity and mortality [1]. The most updated estimates report how, in 2022, lung cancer (LC) was the most commonly diagnosed cancer worldwide, with nearly 2.5 million new cases, accounting for about one in eight cancers (12.4% globally). It was also the leading cause of cancer-related deaths, causing approximately 1.8 million fatalities, which represents 18.7% of all cancer deaths [2,3].

The disease, therefore, represents a significant burden, generating elevated costs and posing a challenge to the sustainability of healthcare systems and services [4,5]. A recent systematic review of 19 studies conducted overall in the world demonstrated that the LC-associated direct mean annual medical costs ranged from USD 4484.13 to USD 45,364.48 (derived using purchasing power parity conversion factor). The total LC-associated medical costs as a percentage of total gross domestic product (GDP) ranged from 0.00248 to 0.1326 (median 0.0217), and the total medical costs as a percentage of total health expenditure ranged from 0.038 to 0.836 (median 0.209), showing how the expenses of LC are substantial and impose a significant economic burden on patients, healthcare systems, and societies [6].

In addition to the high costs associated with LC diagnosis, treatment, and palliative care, lung cancer patients often present with multiple comorbidities that can further complicate clinical management and increase healthcare utilization [7,8]. Therefore, comprehensively capturing the economic burden of lung cancer, including the influence of comorbid conditions, is essential for health policy planning, resource allocation, and cost-effectiveness analysis.

In the realm of healthcare economic evaluation, cost-of-illness (COI) studies aim to estimate the total economic impact of a disease, including direct medical costs such as hospitalizations, medications, specialist visits, and emergency services [9,10,11]. However, many prior studies have not sufficiently accounted for the heterogeneity introduced by comorbidities. In this context, comorbidity phenotyping using data-driven approaches, such as Latent Class Analysis (LCA), identifies distinct multimorbidity patterns that may impact resource consumption differently [12,13].

This study examines the cost of illness associated with lung cancer in a real-world population-based cohort, categorizing patients by comorbidity classes. Both generic healthcare and lung cancer-specific costs were evaluated to quantify the economic impact of the comorbidity burden on resource utilization.

## 2. Materials and Methods

### 2.1. Context

The Italian National Health System is financed through general taxation and administered at the regional level. It operates according to the ethical principles of universal coverage. Healthcare management is supervised by regional authorities, ensuring equitable healthcare services nationwide.

In 2013, the Regional Veneto Government established the Regional Oncology Network (ROV), an interdisciplinary consortium dedicated to providing, implementing, and overseeing diagnostic and therapeutic pathways for oncology patients. In 2022, the ROV published a comprehensive document delineating the PDTA (Italian acronym for diagnostic, therapeutic, and care pathways) for lung cancer patients, encompassing everything from initial diagnosis to end-of-life support [14], grounded in the highest available national and international clinical evidence [15,16,17,18].

### 2.2. Study Design and Population

This retrospective population-based cohort study included 1540 patients with a confirmed lung cancer (LC) diagnosis in 2017 and 2019, across two Local Health Authorities (LHAs) in the Veneto Region of Northeastern Italy. Patients were categorized into five groups based on comorbidity burden: no comorbidities (Comorbidity 0) or the presence of at least one comorbidity without class assignment (Comorbidity 1), and three latent comorbidity classes (Class 1, 2 and 3), derived from a previously performed Latent Class Analysis of administrative and clinical data [18]. The information on the categories of comorbidities was obtained from inpatients’ hospital records reporting the primary and secondary diagnoses classified according to the ICD-9-CM system, as applied at the time of cancer incidence. Only hospitalizations within 6 months of diagnosis were considered. Patients lacking hospital records were excluded from the study. Thirteen primary disease categories were analyzed (presence/absence of major ICD9-CM disease categories and V codes).

### 2.3. Costs Analysis

The cost analysis was conducted using anonymized aggregate data. For both patient cohorts, the cost estimates encompass a three-year period following the initial cancer diagnosis. These estimates account for all disease-related expenses, as provided by the Regional Health Authority. Table 1 outlines the sources and profiles of the aforementioned administrative data. Each patient was assigned a unique and anonymous identification code, which was used to link all administrative data covering hospital admissions, outpatient visits, drug prescriptions, emergency room visits, medical devices, hospice admissions, and vital statuses. The average per-patient costs were calculated and stratified according to the stage of disease at the time of diagnosis.

### 2.4. Statistical Analysis

Descriptive statistics summarized the baseline characteristics by comorbidity group. Statistical significance was tested using chi-square and ANOVA, when appropriate. Latent Class Analysis (LCA) identified patterns of comorbidity. Patients with more than two concurrent diseases were assigned to three latent classes. This method estimates the probability of each individual belonging to a specific class through maximum likelihood estimation criteria; The Akaike Information Criterion (AIC) determined the best number of mutually exclusive and exhaustive classes by comparing several pattern solutions, with the lowest AIC indicating the best fitting model. Consequently, we defined each latent class and assigned it a corresponding name by identifying the most ‘characteristic’ comorbidities—specifically, the top conditions ranked by the percentage distribution of major comorbidities—within each class (see Table 2). The model estimated each individual’s posterior probability of class membership, assigning each person to the class with the highest probability. Tobit regression analyzed the association between the comorbidity class and annual standardized healthcare costs, accounting for administratively censored cost data. Models were adjusted for sex, age categories (<65, 65–75, 76–85, >85), and cancer stage (I–IV, missing).

## 3. Results

### 3.1. Patient Characteristics

The study population (n = 1540) was stratified into five groups: Comorbidity 0 (n = 283), Comorbidity 1 (n = 391), Comorbidity Class 1 (n = 154), Class 2 (n = 229), and Class 3 (n = 483): within Class 1 (Cardiovascular-Respiratory and Endocrine), dominant conditions included diseases of the circulatory system (99.91%) and respiratory diseases (83.16%), as well as endocrine disorders (32.54%). In Class 2 (Multi-organ: Genito-Infectious-Hematologic-Digestive), patients exhibited a higher prevalence of infectious diseases (26.62%), blood disorders (25.22%), digestive diseases (36.11%), and genitourinary diseases (35.31%). In Class 3 (Socio-Multifactorial-Neuro Conditions), the predominant conditions were factors influencing health status (59.27%), symptoms or unspecified conditions (36.93%), and neurological and sense organ disorders (16.71%) (see Table 2).

Significant differences were observed in sex distribution (*p* = 0.038), with males being more represented than females (57.6% vs. 42.4% with no comorbidities, 62.4% vs. 37.6% with comorbidities), and in mean age (*p* < 0.001), with a higher age when comorbidities were present. The cancer stage at diagnosis also differed significantly (*p* < 0.001), with Stage I being more prevalent in patients without any comorbidities and Stage IV, conversely, being less common in this group (see Table 3).

### 3.2. Generic and Lung Cancer-Specific Costs

Overall hospitalization costs increased significantly in patients with comorbidities (*p* < 0.001), as well as outpatient drug and emergency department costs. No significant differences were found in hospice care (*p* = 0.503) and device costs (*p* = 0.132). Instead, a greater cost of inpatient drugs was found in those patients without any comorbidities or at most one (Table 4).

Similar patterns were observed for lung cancer-specific costs: the total lung cancer-specific costs and inpatient drug related costs were significantly different among groups (*p* < 0.001) in those patients without any comorbidities or at most one; conversely, for the outpatient setting, drugs and emergency care-related costs were greater in those with more than one comorbidity (*p* < 0.001). Hospice and device costs did not differ significantly between groups (Table 5).

### 3.3. Tobit Regression Models

The adjusted Tobit model evidenced, in the case of overall costs, that comorbidity Class 3 was significantly associated with higher generic costs (Coeff: 0.601, *p* = 0.008), while Class 1 and 2 were not substantially different from those with no comorbidity; a higher cancer stage (II–IV) was also a strong predictor of increased costs (all *p* < 0.001), while a higher age starting from >60 was associated with lower overall costs (Table 6); also for lung cancer-specific costs, only Class 3 was significantly associated with higher costs (Coeff: 0.498, *p* = 0.078), though the effect was marginal; advanced age and cancer stage were significant cost drivers (Table 7).

## 4. Discussion

This study highlights the significant impact of comorbidities on healthcare costs in lung cancer patients, underscoring the importance of considering comorbidity profiles in economic evaluations.

Our results are consistent with prior studies documenting the influence of comorbidities on cancer patients’ pathways [19,20] and healthcare costs [21]. A recent study [22] examining medical costs by lung cancer stage and histology found that advanced disease stages, often accompanied by multiple comorbidities, are associated with higher healthcare expenditures. Also, another recent study [23] demonstrated that comorbidity status is strongly correlated with hospital charges for lung cancer patients. Specifically, patients with diabetes, hypertension, and both conditions incurred higher hospital charges compared to patients without comorbidities [24]. Furthermore, diabetes may adversely influence survival outcomes and contribute to complications in patients with lung cancer [25]. Another investigation, which analyzed various aspects of healthcare costs, revealed that comorbidities among lung cancer patients limit treatment options and impose a significant additional burden on healthcare resources. This study involved 8655 patients with lung cancer, of whom 31.3% had at least one comorbid condition; the presence of comorbidities was associated with increased annual and inpatient expenditures [26].

However, our study showed that having more than one comorbidity was linked to a lower cost of inpatient treatments, such as chemotherapy. The possibility that comorbid conditions influenced the treatment choice is not, however, standardized, as comorbidity was still absent in the latest Veneto Region pathways for the clinical diagnosis and treatment of lung cancer [14]. These pathways predominantly base the selection criteria for surgery, chemotherapy, radiotherapy, and targeted therapy on lung cancer stage and genetic testing. However, clinicians may be concerned that there is a higher risk of treatment toxicity, side effects, and complications associated with comorbidity, or that the supposed reduced life expectancy of patients with comorbidity may dissuade physicians from pursuing aggressive treatment options [27,28]. Furthermore, there exists scarce high-level evidence concerning the effects of cancer therapies in patients with comorbidities, considering that randomized controlled trials frequently exclude individuals with concomitant severe conditions. This paucity of evidence further constrains clinicians’ decision-making and often leads to the adoption of more conservative treatment approaches [7]. Our findings underscore the need for research on curative therapy among patients with comorbidity and the development of treatment decision aids that incorporate the impact of comorbidity on survival and quality of life for clinicians. They also highlight the potential challenges of comorbidity in the management of lung cancer treatment. Integrating comprehensive comorbidity assessments into clinical practice can facilitate the development of personalized treatment plans that address the specific needs of patients with complex health profiles. Moreover, policymakers should consider these variations in comorbidity-related costs when allocating healthcare resources and designing interventions to enhance outcomes for patients with lung cancer. 

In addition, our study found differences among comorbidity patterns; in particular, our results showed that patients categorized in Comorbidity Class 3 (Socio-Multifactorial-Neuro Conditions) were generally older and had more advanced disease stages than other groups. However, after adjusting for age and stage, these patients still incurred higher overall healthcare costs. A prior study indicated that possessing a prescription for two or more classes of psychotropic medications for a minimum of 90 days within the first year following a cancer diagnosis was correlated with an increased frequency of outpatient visits, office consultations, hospital admissions, and extended lengths of stay [29]. The elevated costs associated with Class 3 may be attributed to the multifaceted care requirements of patients with socioeconomic challenges and neuropsychiatric disorders, which often necessitate comprehensive and prolonged medical interventions [30]. Instead, greater disinvestment in hospital drugs was found in class patients who are affected by multi-organ diseases, and this is likely due to the increased risk of side effects in cases of comorbidities.

### Strengths and Limitations

A strength of this study is the application of an LCA-derived classification to categorize comorbidities, which offers a nuanced understanding of how various comorbidity patterns influence costs. Limitations include the retrospective design and the potential for residual confounding factors not accounted for in the analysis. Furthermore, a limitation of the study is its reliance solely on hospital discharge records for the evaluation of comorbidities, without the inclusion of all administrative databases. By restricting the comorbidity assessment to hospital discharge data, the study may have overlooked certain comorbidities managed in outpatient settings or those not severe enough to necessitate care and consequently be recorded during hospital admissions. This approach could potentially result in an underestimation of the actual comorbidity burden and its influence on healthcare costs for some patients. Finally, this study not adjusting for these socioeconomic and marital status factors is a limitation, as they represent potential confounding variables that could influence both comorbidity patterns and healthcare expenditures in this lung cancer population. Future research should aim to incorporate these sociodemographic variables to provide a more comprehensive understanding of how they interplay with comorbidities to impact healthcare costs among cancer patients. However, by incorporating these V codes when defining the comorbidity classes through latent class analysis, the study was able to partially capture information, serving as a proxy, related to socioeconomic determinants that may influence healthcare utilization and costs.

## 5. Conclusions

This study is the first to document how distinct comorbidity classes significantly impact healthcare costs in patients with lung cancer. Recognizing and addressing these differences are crucial to optimizing care delivery and resource utilization in this vulnerable population, offering valuable insights for policymakers and healthcare providers to better identify patients with elevated healthcare needs by evaluating their comorbidity profiles, thereby enabling the more effective allocation of lung cancer care resources. Additionally, the findings may support the creation of more integrated disease management strategies aimed at enhancing patients’ quality of life while simultaneously addressing cost efficiency.

## 6. Clinical Practice Points

Lung cancer poses a challenge to the sustainability of healthcare systems.

Cost items differ based on comorbidity groups. The overall costs (both lung cancer-specific and non-specific) for hospitalizations, emergency room visits, and outpatient medications increased among patients with comorbidities; however, higher costs for inpatient medications were noted in patients without any comorbidities or with at most one.

The patients with only one comorbidity class were linked to the highest overall and lung-specific costs.

Lung cancer-specific expenses were lower in patients with more than one comorbidity group. The lowest overall expenditures for lung cancer were observed in the Cardiovascular-Respiratory and Endocrine class.

## Figures and Tables

**Table 1:** Healthcare costs of NCLSC patients; administrative regional databases included in the cost estimates.

Administrative Databases	Data Collection
Hospital admissions	Defines the Diagnosis-Related Groups (DRGs) for each admission, assigned based on an inpatient formulary (such as Tariffario Prestazioni Ospedaliere), covering all hospital activities.
Outpatient visits	Procedures and services offered at outpatient facilities funded by regional health services. The economic values are determined according to rates set by an outpatient formulary, such as the Tariffario Prestazioni Ambulatoriali.
Emergency room admissions	The costs are calculated according to the rates applicable to all medical procedures and interventions conducted during Accident and emergency visits.
Pharmaceutical distribution and hospital drug consumption	Consider the expenses associated with medical therapies based on prescribed dosages. Component drug delivery costs refer to medications administered by hospital pharmacies but not included in the hospital admission charges.
Community drugs	Consider the expenses associated with medical therapies based on prescribed dosages. Component drug delivery costs refer to medications administered by outpatient pharmacies.
Medical devices	Reports the expenditures incurred by regional authorities for the provision of medical devices.
Hospice admission	Costs are determined by multiplying a regional daily rate by the number of days spent in hospice.

**Table 2:** Percentage distribution of comorbidities (the class in which each comorbidity occurs most frequently is highlighted in bold) among latent Class 1, 2, and 3. Class 1 includes Cardiovascular-Respiratory and Endocrine diseases; Class 2 includes Multiorgan diseases; Class 3 includes Socio-Multifactorial Neuro Conditions.

ICD9-CM Disease Category Classification	CLASS 1 Cardiovascular-Respiratory and Endocrine Diseases	CLASS 2 Multiorgan Diseases	CLASS 3 Socio-Multifactorial Neuro Conditions
Infectious and Parasitic Diseases	0.01%	**26.62%**	2.35%
Endocrine, Nutrition, Metabolism, Immune Disorders	**32.54%**	26.91%	20.79%
Blood and Hematopoietic Organs	8.78%	**25.22%**	15.76%
Mental Disorders	7.16%	6.11%	**8.26%**
Nervous System and Sense Organs	6.86%	6.61%	**16.71%**
Circulatory System	**99.91%**	52.57%	51.44%
Respiratory System	**83.16%**	45.34%	56.93%
Digestive System	3.44%	**36.11%**	9.58%
Genitourinary System	19.12%	**35.31%**	10.13%
Musculoskeletal and Connective Tissue	4.53%	**15.08%**	12.51%
Symptoms, Signs, and Undefined Conditions	10.48%	25.74%	**36.93%**
Injuries and Poisoning	1.08%	**19.99%**	17.32%
Factors Influencing Health Status (V codes)	1.90%	29.58%	**59.27%**

**Table 3:** Demographic and clinical characteristics by comorbidity group.

	No Comorbidity (n = 283)	1 Comorbidity (n = 391)	Class 1 (n = 154) Cardiovascular-Respiratory and Endocrine Diseases	Class 2 (n = 229) Multiorgan Diseases	Class 3 (n = 483) Socio-Multifactorial Neuro Conditions	*p*-Value
Sex n (%)						0.038
F	120 (42.4%)	147 (37.6%)	47 (30.5%)	71 (31.0%)	169 (35.0%)	
M	163 (57.6%)	244 (62.4%)	107 (69.5%)	158 (69.0%)	314 (65.0%)	
Mean age (DS)	71.4 (11.3)	74.1 (11.2)	77.8 (9.8)	76.5 (9.2)	73.5 (10.8)	<0.001
Age class n (%)						<0.001
<65 anni	67 (23.7%)	77 (19.7%)	15 (9.7%)	26 (11.4%)	102 (21.1%)	
65–75 anni	103 (36.4%)	128 (32.7%)	40 (26.0%)	72 (31.4%)	153 (31.7%)	
76–85 anni	87 (30.7%)	121 (30.9%)	62 (40.3%)	85 (37.1%)	159 (32.9%)	
>85 anni	26 (9.2%)	65 (16.6%)	37 (24.0%)	46 (20.1%)	69 (14.3%)	
Stage n (%)						<0.001
I	50 (17.7%)	43 (11.0%)	7 (4.5%)	15 (6.6%)	50 (10.4%)	
II	23 (8.1%)	19 (4.9%)	6 (3.9%)	9 (3.9%)	25 (5.2%)	
III	45 (15.9%)	49 (12.5%)	15 (9.7%)	31 (13.5%)	81 (16.8%)	
IV	153 (54.1%)	269 (68.8%)	123 (79.9%)	166 (72.5%)	318 (65.8%)	
Missing	12 (4.2%)	11 (2.8%)	3 (1.9%)	8 (3.5%)	9 (1.9%)	
Overall survival at three years after diagnosis	109 (38.5%)	95 (24.3%)	15 (9.7%)	41 (17.9%)	85 (17.6%)	<0.001

**Table 4:** Three-year generic healthcare costs (all cause) by comorbidity category (€: mean per patient).

	No Comorbidity	1 Comorbidity	Class1 Cardiovascular-Respiratory and Endocrine Diseases	Class 2 Multiorgan Diseases	Class 3 Socio-Multifactorial Neuro Conditions	Overall	*p*-Value
Hospitalizations	13,028.54	13,666.04	13,246.98	15,539.10	17,719.42	15,000.21	<0.001
Hospital drugs	22,996.38	24,825.12	15,651.43	13,483.54	15,798.49	19,627.14	0.077
Community drugs	1117.88	1245.28	1455.04	1451.11	1713.92	1370.89	0.001
Outpatient	8352.54	9207.73	5479.90	6843.54	8531.85	8162.09	<0.001
Emergency room	497.02	517.20	676.61	749.27	761.72	630.56	<0.001
Hospice	701.76	908.00	1208.46	761.19	1156.79	929.63	0.503
Medical devices	995.93	1670.48	728.69	1433.49	1701.26	1434.79	0.132
Total costs	47,690.06	52,039.85	38,447.09	40,261.25	47,383.45	47,155.30	<0.001

**Table 5:** Three-year lung cancer-specific healthcare costs by comorbidity category (€: mean per patient). * Note: For these cost categories, no specific lung cancer-related costs were defined. So, overall values were used.

	No Comorbidity	1 Comorbidity	Class 1 Cardiovascular-Respiratory and Endocrine Diseases	Class 2 Multiorgan Diseases	Class 3 Socio-Multifactorial Neuro Conditions	Overall	*p*-Value
Hospitalizations	10,256.53	10,213.47	10,625.72	10,625.65	13,014.59	11,167.62	<0.001
Hospital drugs	22,658.12	24,620.53	13,494.66	12,941.40	15,433.79	19,201.90	0.026
Community drugs *	1117.88	1245.28	1455.04	1451.11	1713.92	1370.89	0.001
Outpatient	7970.72	8736.32	4943.81	5590.79	7996.57	7608.88	<0.001
Emergency room *	497.02	517.20	676.61	749.27	761.72	630.56	<0.001
Hospice	690.57	800.82	1207.10	633.75	1066.04	856.31	0.466
Medical devices *	995.93	1670.48	728.69	1433.49	1701.26	1434.79	0.132
Total costs	44,186.76	47,804.09	33,131.63	33,425.46	41,687.90	42,270.95	<0.001

**Table 6:** Tobit regression overall healthcare costs (coefficients in € 000).

	Variable	Coef. (€ 000)	SE	95% CI	*p*-Value
	(Intercept)	10.85	2.26	(6.42; 15.28)	<0.001
Comorbidity (ref = 0)		1.00			
	One comorbidity	−0.80	1.68	(−4.08; 2.48)	0.634
	Class 1	−2.30	2.18	(−6.57; 1.98)	0.293
	Class 2	1.01	1.92	(−2.76; 4.78)	0.601
	Class 3	4.26	1.61	(1.10; 7.41)	0.008
Sex (ref female)		1.00			
	Male	1.10	1.14	(−1.13; 3.34)	0.332
Age (ref age class < 44)		1.00			
	Age 45–59	0.17	1.59	(−2.94; 3.28)	0.913
	Age 60–74	−5.82	1.59	(−8.94; −2.71)	<0.001
	Age ≥ 75	−12.51	1.91	(−16.25; −8.77)	<0.001
Stage (ref stage I)		1.00			
	Stage II	11.47	2.89	(5.80; 17.14)	<0.001
	Stage III	16.80	2.21	(12.46; 21.14)	<0.001
	Stage IV	19.11	1.84	(15.51; 22.71)	<0.001
	Missing	9.81	3.73	(2.51; 17.12)	0.008

**Table 7:** Tobit regression for lung cancer specific costs (coefficients in € 000).

	Variable	Coef. (€ 000)	SE	95% CI	p-Value
	(Intercept)	9.36	2.04	(5.36; 13.37)	<0.001
Comorbidity (ref = 0)		1.00			
	One comorbidity	−0.89	1.52	(−3.86; 2.08)	0.558
	Class 1	−2.93	1.98	(−6.81; 0.95)	0.138
	Class 2	−1.18	1.74	(−4.60; 2.24)	0.498
	Class 3	2.57	1.46	(−0.29; 5.42)	0.078
Sex (ref female)		1.00			
	Male	0.74	1.03	(−1.28; 2.77)	0.471
Age (ref age class < 44)		1.00			
	Age 45–59	0.31	1.44	(−2.51; 3.13)	0.829
	Age 60–74	−4.76	1.44	(−7.58; −1.93)	<0.001
	Age ≥ 75	−10.98	1.73	(−14.37; −7.59)	<0.001
Stage (ref stage I)		1.00			
	Stage II	10.60	2.62	(5.46; 15.74)	<0.001
	Stage III	15.96	2.01	(12.03; 19.89)	<0.001
	Stage IV	18.69	1.66	(15.43; 21.95)	<0.001
	Missing	9.65	3.38	(3.03; 16.26)	0.004

## Data Availability

The data supporting the findings of this study are held by the Veneto Epidemiological Registry and were used under license for the present work, but they are not publicly available. These data are nonetheless available from Manuel Zorzi on reasonable request and subject to permission to be obtained from the Veneto Epidemiological Registry (Veneto Regional Authority).
